# Gold as a Filling Material

**Published:** 1900-07-15

**Authors:** N. S. Hoff


### GOLD AS A FILLING MATERIAL.

BY N. S. HOFF, D.D S.

By way of introduction, may we not first inquire why gold is so highly valued as a filling material? We may properly give it first place for esthetic reasons.

The color is such as will harmonize with all complexions and most shades of teeth. The color is durable, and if by chance it should become tarnished, it is superficial and readily renewed. It is capable of being made very smooth, so that it may be kept from the contaminating influences of the mouth.

It does not change form under the strain of mastication, nor dissolve in the fluids of the mouth. Because of its beauty and indestructibility it is not without a wholesome effect on the mental and moral nature of the patient who wears it in his teeth. In consequence he believes himself less base, and makes greater efforts to deport himself in a decent and becoming manner, so far as his mouth is concerned, at least he keeps his mouth clean. His associations with this pure and noble inanimate substance has much the same effect upon him as an intimate association with a pure and noble friend would have. Because of its lack of immoral tendency and its intrinsic purity and stability it has a strong claim for first place on purely ethical grounds. If it exerts an uplifting influence on one who wears it in his mouth, what shall we say of one whose business it becomes day after day to so study its characteristics that he may advantageously use them for so noble a purpose as that of bettering the physical condition of mankind?

We, as dentists, find we can bend it, hammer it, roll it into plate, draw it into wire, shape it to beautiful forms which we can polish, and, by so doing, still further enhance its beauty. And yet, from our experience with it, we find that it makes imperative demands when we attempt to utilize its excellent qualities.

We find that to make it workable we should not contaminate it with baser metals. We must anneal it and take advantage of certain qualities and observe well-known principles or it becomes unmanageable. And so we find that whoever would best utilize its splendid virtues, must subdue himself and become the servant for the time, that he may attain the credit of producing from this noble metal something beautiful and useful. Each of us have undergone a long and patient course of training to reach even a moderate degree of skill in filling teeth with this noble metal.

Can we appreciate as we should the mental and moral value of this self-denial? So far as we do, I am sure there is no subject nor substance associated with our training that has had more to do with the cultivation of a high standard of morals and technical attainments than has come as the direct result of our effort to utilize this material.

As evidence of its reputation, may I call your attention to the following quoted extracts from well-known text-books as to its use in only one department of our profession. In regard to its use as a material for filling teeth, Dr. Taft, in an early addition of his work on "Operative Dentistry," (page 86) says: "Of all metals used for filling teeth, gold possesses more requisites than any other."

Dr. Louis Jack, in the American Text-Book, (page 168) says: "Gold possesses the highest preservative qualities, and promises greater durability and more satisfactory results than any other material."

Dr. Gorgas, in Harris' "Principles and Practice," twelfth edition, (page 499) says: "To the use of gold, when properly prepared, there is the least possible objection; a tooth may be so filled with it as to secure in almost every case its permanent preservation."

Dr. Fillebrown, in his book, (page 68) says: "Gold is the most elegant material for filling teeth that we possess, and for most cases it is the best."

Dr. Ottolengui, in his book, (page 63) says: "Gold is pre-eminently the best material with which to fill teeth, and our main reliance for the salvation of teeth which have decayed must be upon gold."

On the contrary, we do not perhaps discredit the above statements in quoting the only strong contradictory statement. That familiar quotation from Professor H. S. Chase, endorsed by Professor J. Foster Flagg in his work on "Plastic Fillings," (page 23) as follows: "In proportion as teeth need saving, gold is the worst material to use."

May we not justify or enlighten our own convictions as to the place and comparative value of gold as a good material with which to fill teeth by subjecting it to the classical standards maintained by all recognized writers on the subject of filling materials?

Will it stand the strain of mastication? Undoubtedly no material that we now use can approach it even in this respect. It is perhaps not so hard as amalgam, its nearest competitor, still it is tougher and more resistant, especially in those cases where the edges are thin or where small bands connect larger masses of filling material. Amalgam being hard and brittle is liable to break, while gold, even in small quantities, will withstand great stress without giving away, demonstrating its superiority in this particular at least.

Does it, when carefully and properly applied, thoroughly seal the cavity and protect the denuded dentin from recurring decay?

This involves the question of adaptability, and probably for this reason gold is surpassed by almost all other materials used for filling teeth. Amalgam, gutta-percha, cements, and possibly tin foil, are all more easily applied or adapted to the tooth cavity; and yet, while these materials surpass gold in ease of adaptation, they suffer in another important particular which is essential. They have not the same endurance, and therefore do not provide permanent adaptation and protection. Because of molecular dissolution, either from physical or chemical reactions, which are continually taking place in the mouth, the substances mentioned fail to protect the cavity from the reappearance of decay-producing agencies. And gold, although more difficult of adaptation by providing a more permanent sealing of the cavity than any other material, more fully conforms to even this standard. Gold certainly withstands much better than any other material the onslaughts of acids which destroy the baser metals and alkalies which disintegrates the acid cements.

Is it compatible with the sensitive tooth structure?

In one respect gold suffers badly at this test. It responds readily to thermal changes, having a higher potential in this respect than any other filling material.

As conductors of heat we may grade filling materials in the following order: Gold, amalgam, tin, cement, gutta-percha.

Because of this undesirable quality, gold sometimes proves to be a very troublesome irritant. Gold itself, however, is kindly tolerated by sensitive dentin because it is not readily soluble or oxidizable. As an insoluble substance it is practically inert, and of itself the least irritating material which can be placed upon sensitive dentin. It sometimes happens that by approximate contact with filling materials of amalgam or tin it induces electrical disturbances which may result in irritating magnetic currents in the region of the fillings, resulting possibly in the disintegration of the tooth itself. This is, however, an incidental phenomena rather than an accompanyment of gold fillings. Most other materials, especially the cements and amalgams, because of their greater solubilities, are more likely to prove incidentally incompatible with the dentin than gold.

As a result of this examination of gold as a filling material, need we any further evidence to establish its claim to superiority?

This preliminary consideration of the qualities of gold as a filling material has, I trust, prepared us to consider the various preparations of gold which not only adapt it to this purpose, but render difficult operations comparatively easy.

In spite of the fact that during the past few years great interest has been centered in the exhaustive researches of Dr. Black and others which were designed to ascertain the cause of the physical change and disintegration which render amalgam a somewhat unreliable and unsatisfactory filling material, there has probably never been a time when greater interest was shown in efforts designed to provide preparations of gold which would conserve all its excellent qualities, and render it more easy of adaptation. We rejoice that these efforts have met with some measure of success.

The gold manufacturers have exercised themselves as never before to provide more elegant preparations, and the result is that gold of many forms may be had, each of which has a special claim for excellence. One preparation making the claim that it is so well adapted to the purpose that it almost fills the tooth itself.

There are, however, but two kinds of gold used in filling teeth, cohesive and non-cohesive. I have been sufficiently curious to know what relative amounts of each kind were being manufactured and sold that I sent to four of the largest manufacturers and dealers in gold foil in this country and asked them to give me an estimate of the relative amount of each kind of gold they sell. Of course, the best reply I could get was an estimate. I was considerably surprised to find how nearly the replies corresponded in the important particulars. The two classes of gold I mentioned were cohesive and non-cohesive. But as some manufacturers make gold preparations which may be used when annealed as cohesive and when unannealed as non-cohesive, I therefore made my classification under three heads, and below I tabulate the replies I received from the manufacturers.

| MANUFACTURER. | NON-COHESIVE. | COHESIVE. | EITHER. |
|---|---|---|---|
| A. | 5 percent. | 65 percent. | 30 percent. |
| B. | 5 " | 70 " | 25 " |
| C. | 10 " | 80 " | 10 " |
| D. | 1/3 " | 99 2/3 " | 0 " |
| Total averages, | 5 1/4 " | 78 1/2 " | 16 1/4 " |

From this table it will be seen that a very large percent (78 1/2) of the gold used in filling teeth is cohesive and a very small percent (5 1/4) is non-cohesive.

The probabilities are that the percentage of cohesive gold would be very much increased because of the use of the "either percent," (16 1/4) very much of which is doubtless used as cohesive foil.

This demonstration would seem to indicate that efforts to render gold more pliable and more easily manipulated are not being sought in the direction of non-cohesive foil, as we would be inclined to infer from the fact that it has always been the claim for non-cohesive foil that because of lack of cohesion it could be more easily and thoroughly packed into the angles and undercuts of comparatively inaccessible cavities. On the contrary, it would seem to suggest either a renewed interest in endeavors to restore contours or a more general practice of this method of filling than is generally credited.

We may not be justified in drawing these conclusions from this data because of its incompleteness, and also because of the fact that non-cohesive foil filling is scarcely taught at all in the dental schools. Consequently the new men in the profession are prejudiced in favor of cohesive foil. Still our conclusion may be justified by another fact, namely, that the more recent preparations of gold are made to be used as cohesive gold, although some, when unannealed, can be used on non-cohesive principles.

There are in use to-day three forms of the two kinds of gold most of which are intended to be used cohesively, but some of them can not be used cohesively by any treatment.

The first form would include the foils of every kind which come into the hands of the dentist in the shape of sheets of varying thickness but uniform in size, containing varying amounts of gold, indicated by a number. The No. 2 foil, for instance, is a sheet about four inches square containing two grains of gold, while No. 60 is a sheet of the same size containing sixty grains of gold; in other words, No. 60 foil is thirty times as thick as No. 2. The thinner foils, Nos. 2 to 6, are generally used by rolling them into ropes and cutting these ropes to varying lengths, from one to two inches, making pellets or rolls. The heavy foils are cut into narrow strips or ribbons from one-eighth to one-fourth inch in width and from one to two inches in length. The thinner foils may be used on the cohesive principle by annealing, and when not annealed may be worked as non-cohesive foil. The thicker foils are always annealed at the time of their introduction into the cavity.

There are objections to the manipulations of the thinner foils by the operator who prepares it extemporaneously by rolling it into ropes with a napkin or a piece of punk as it is often done. Unless done with great care the foil is not in the best form to produce a solid filling. By careless manipulation the foil is crumpled so much as to produce great inequality in the amount of gold in each pellet or rope, so that in annealing and packing there is a lack of uniformity in the density of the filling. If this manipulation has been done in a slovenly manner there is likely to be incorporated sufficient extraneous matters to interfere with the union of the several layers of foils, and the filling in time will tarnish and disintegrate because of imperfect welding. The preparation of foil into pads which are then cut into strips or pellets is a cleaner and more preferable method as it places the sheets in smooth and regular form and impurities are less likely to be incorporated. This also facilitates packing and produces a more solid filling.

In consolidating pellets cut from a rolled rope, because of the irregularity of their structure it is difficult to drive out the enclosed air, which prevents complete consolidation, and also prevents that close adaptation to the tooth walls which is essential to a good filling.

The heavier foils are ideal in this respect. Their preparation is very simple. The foil is cut into suitable shaped ribbons of varying sizes, which are easily and perfectly annealed with little danger to the foil's integrity.

This foil is so cohesive that its adaptation requires only care to place it with the plugger where wanted. It has the advantage of having only two surfaces to weld, where the same amount of gold in thinner foil or pellets would have from ten to twenty surfaces perhaps. It can be manipulated with smaller instruments and less force in malleting to secure the same and even closer union.

This form of gold not only produces a substantial filling throughout, but provides a surface which will take a high finish and remain so under the most severe use to which it may be subjected showing little or no appreciable change in its form or surface. It will also retain its position with more shallow anchorages.

In locations where it is necessary to carry it over exposed borders or through narrow fissures it is invaluable, because the full tensile strength of wrought gold in its strongest form is secured. Repairs to fillings made with this form of foil can be securely made because there is less liability to dislodge the original filling and secure anchorages may be made in its substance. In some respects a high order of skill is required for its manipulation, but reasonable care only may be exercised to avoid the tendency to stuff the cavity rather than to fill it.

With proper facilities and conscientious attention to details, tooth filling with this form of foil may be done as expeditiously as with any other.

Pellets and cylinders, while properly constituting a form of gold, are, in fact, only modified preparations of foil, and are generally made by foil manufacturers, although sometimes extemporaneously made by the operator.

The lighter rolled forms are likely to deteriorate in transportation and by being kept in stock, hence the operator many times prefers to manufacture for himself. But the process requires such nice manipulation and so much time that most operators prefer to depend upon the manufacturer's article. They are made from the thinner foils of such shapes and sizes as will meet the demands of the different systems of operating. The effort is to supply the gold in a convenient form for ready introduction into the cavity and to make it uniform in structure so as to facilitate welding.

The cylinders are admirably adapted to filling proximal cavities in molars and bicuspids, also some classes of crown cavities; especially useful with the so-called "hand pressure" method. But their greatest value is to be found in the combined hand pressure and mallet. The use of cylinders by these methods will result in good fillings easily and quickly made. Like other good things the pellets and cylinders when properly used produce satisfactory results, but when ignorantly or carelessly employed may cause disastrous results. The chief fault lies in the failure to properly place and condense each pellet or cylinder. They may be used without annealing, and when so used can be wedged latterly against the walls of the cavity or into angles and undercuts to some extent. But when annealed they should be condensed by direct pressure where placed, and should not be expected to change position to any considerable extent. Failure to recognize this distinction will result not only in imperfect condensation but lack of cohesion, and possibly of imperfections in adaptation.

These preparations should be annealed with great care, as there is great probability that by direct exposure to the flame the pliable nature of the gold may be destroyed. They should also be handled or preserved with more care than is customary. They should be kept from exposure to the air and carefully handled so as not to change their mechanical structure.

The third form of gold preparations for filling teeth includes the precipitated. or crystal, or "mat golds." In 1853 Dr. A. J. Watts, of Utica, N. Y., took out a patent for making the first crystal gold—it was, however, discovered and first made by Dr. H. R. White, of Utica, N. Y., in 1851. It was then called sponge gold. It was made by dissolving pure gold in nitro-muriatic acid and precipitating it with oxalic acid in such a way as to induce the precipitate in the form of long crystals or fibres. The addition of mercury and heat was used to control the precipitation and determine its character.

The earlier preparations of crystal gold, although crude, met with much favor, and were partially abandoned when cohesive gold foil was introduced, and recently to again engage our attention because of very much improved preparations, made by modified processes. We have at the present time several excellent preparations of crystal gold, all of which may be classified as cohesive, although without being annealed they are sufficiently non-cohesive to be worked on that principle.

They are, when properly annealed, capable of condensation into a solid mass, which may be rolled into plate. Contour fillings capable of taking and retaining a high polish are also made, and crown cavities which receive hard wear show little or no sign of destruction after long use. I have, however, noticed that thin edges where much exposed have a tendency to break out, indicating either brittleness or lack of complete welding.

I have made considerable use of this form of gold during the last three years with quite satisfactory results. For very small proximal fillings in the incisors, and also in other teeth the cavities of which may be in somewhat inaccessible positions, or such as would require extensive loss of tooth structure to prepare the tooth for ordinary filling material it is almost invaluable. Instead of cutting these cavities through to the occlusal or labial surfaces, they may be opened labially or lingually and the cavity made sufficiently retentive in form to receive a considerable piece of the crystal or mat gold, which can then be consolidated with hand pluggers of suitable angles to reach the grooves. The entire cavity is oftentimes best filled with the same preparation of gold, yet I find it often possible and expedient to finish the filling with some other preparation of foil.

The greatest satisfaction I have secured from it, however, came from using it to start fillings. Very little retaining shape is needed in many cases, as this gold seems to adhere to the tooth substance, and particularly if a sharp corner or angle is made into which the first piece may be driven. It will settle itself with slight manipulation definitely and securely, so that subsequent additions of the same or other forms of gold may be successively added without displacing it. By the use of this form of gold for such purposes deep undercuts and retaining points are avoided, and in many cases of frail or sensitive teeth this is quite desirable.

I find it of great service in starting fillings in the proximal surfaces of bicuspids and molars which involve the entire surface and extend beneath the gum margin, necessitating the use of the matrix. It is almost always difficult to make a suitable seat for fillings in such cases. But with a suitably adjusted matrix and a slight groove or undercut on the lateral walls it is easy to start such fillings and make a secure joint at the cervical margin, and because of the ready adaptation of the gold, good lateral joints are also possible. My practice is to carry such filling up into the cavity as far as may be necessary with alternate layers of mat gold and No. 60 foil until I can obtain direct access and a clear view for the manipulation of the heavy foil, completing the filling with the No. 60 foil.

In these cases I secure a strong anchorage for my filling on the occlusal surface of the tooth, and by building a considerable part, say one-third, of the filling with No. 60 foil I feel confident that no amount of strain from mastication, which the tooth itself can stand, will disturb the filling.

It is my judgment that we have in this preparation of gold an invaluable helper.

It is a great time saver, it will enable us without great fatigue to fill with gold cavities which we are inclined to trust to the unreliable cements or amalgams, or to other unsatisfactory expedients.

This gold needs careful handling. It should be carefully annealed, carefully manipulated and used with skill and good judgment.

It will not work itself, and it is not the best preparation for all cases.

I certainly believe, however, that it will be the means of stimulating new interest in the best known material for filling teeth.

DISCUSSION.

The discussion was opened by DR. WATLING, of Ypsilanti.

*Mr. President, Ladies and Gentlemen:*—I wish some other member of the Association who is in a better talking mood than myself had been called upon to open this discussion.

Dr. Hoff has submitted one or two questions for me to comment upon.

Has non-cohesive gold any considerable value as a filling material?

In my opinion it has. In speaking of the non cohesive gold in the sense that he mentions here to-night I do not know that I ever handled any, I do not know that I have ever used any gold which I could not render cohesive by annealing, I presume that it has been my fault, because I never bought it. I do not remember of ever using that kind of gold. I started in the profession something over forty years ago, and at that time cohesive gold was a new thing.

I really never learned the art of using gold except as cohesive gold with serrated pluggers. I can put in a small crown cavity at the present time with the old style of filling with sharp pluggers and strictly non-cohesive gold, but if it be a large cavity I know I could not fill it as I did years ago.

For many years in my practice I used nothing but the most strictly cohesive foil that was made, Watt's crystal gold foil. I do not know whether it is manufactured at the present time or not. It was the most cohesive of all gold preparations.

Question: Did you say that it was a foil?

Dr. Watling: It was a foil.

The first piece of gold I put into a cavity I never anneal. It is practically non-cohesive gold, but I anneal each succeeding piece. The amount of annealing depends somewhat upon the manufacture of the gold. If I find that the gold tends to break in working I would anneal it more and more as the filling progresses. I do not know that I can answer this question as to strictly non-cohesive gold, as I am not sufficiently acquainted with its use. To illustrate: My system in starting a filling, taking a large approximate filling in a bicuspid; I would commence at the cervical, the first piece of gold being sufficiently large to cover the bottom of the cavity, extending out over margin of cavity quite freely so the first piece extends out over the edge and fills that place where fillings so frequently fail, at the cervical border. I would rather that the first piece be just as soft and pliable as it is possible to have gold made, yet it must be in this condition so the next piece applied will cohere. It is not necessary to have this first piece condensed absolutely hard.

The old operators paid no attention to the cohesiveness of gold and they made excellent fillings, their fillings were made simply on the principle of corking a cavity, and that is really the true idea. It would be very difficult to cork a beer-bottle, so as to keep the gas from escaping, with a piece of hard wood, it must be a soft and yielding substance which will adapt itself to the opening; just so with fillings, the softer the material used the better it will adapt itself to the walls of the cavity. I call this gold I use non-cohesive or soft gold. According to Dr. Hoff's statement the annealing of the soft gold should render it more soft, but when it is annealed it becomes stiff and when placed in the cavity it is not likely to slide upon itself in malleting.

Has the form in which the gold is used any particular advantage in itself, which can not be overcome by patience and acquired skill on the part of the operator? That is, can not any form of gold be used by different operators and accomplish the same results?

I think that this question can be answered affirmatively. I think we use these different golds somewhat automatically or as a matter of habit. The man who uses exclusively one kind of gold may make just as good a filling as another who uses an entirely different kind; one may use No. 60, another No. 4. A man may perhaps make just as good fillings by the use of sponge gold if he takes just as much or a little more care perhaps, as the one who depends upon foils. We listen to these men who are selling this sponge gold and they tell us how much time they save, about one-half or three-fourths, and they take up large pieces and put in with large pluggers. Now, that is a great mistake, and that is the reason why we have epidemics of sponge gold for two or three years and then it is dropped, it has been having a run within the past five years; men have been showing off their sponge gold, but I see they are not here at this meeting.

It requires just as much pains and care to make a good filling with sponge gold as with any other. There are millions of little infinitesimal particles to be welded together to make such fillings, while the foils, being already welded together, do not require as careful and slow manipulation. In Chicago, two years ago, one of these agents was showing off his crystal gold and making a good deal of talk. He had a tooth imbedded in a piece of lead, and was putting in large pieces and talking about the ease of operating, digging it with a knife to show its hardness. I said: "Let me see that, please." He offered me the knife, saying: "Take it and see how hard it is." I replied: "No, sir; I have a little instrument here of my own." [Laughter.] I could pass the instrument all around the edge of that filling, it was so soft. He had made no consolidation against the walls of the cavity; the filling in the centre was hard. It is the perfect adaptation to the borders which makes the good filling.

As to the use of the heavy golds, there is no question that good fillings can be made with them. It is simply a matter of custom. I have used heavy foils, and do so now in many cases, but it is very seldom I use the heavy foil to start with. I take something lighter as a starter and end up with the heavier. The heavy foil is like a piece of gold plate; it is almost plate. In my hands it requires a good deal of care, and it is not so easily adapted to those fine inequalities of the cavity as the thinner foils. I can not start a filling so readily, either, as with the thinner and softer gold.

The essayist speaks of using sponge gold as a starter; I never have any trouble starting a filling with foil. I have students who have such trouble, and, after getting the first piece in place, they can not add the second. Just like a boy learning to ride a bicycle; he has trouble at first, but soon learns.

When I get up out of the woods and have the gold thoroughly condensed against the walls, I do think that then the No. 60 foil makes the ideal filling. Using fine pluggers, it will give a better surface and more lasting finish, perhaps, than any other foil.

The essayist has enlarged considerably on the subject of the preparation of the gold, whether it shall be rolled in napkins or fingers, or whether it should be laid out in a nice flat ribbon form. That is a matter of theory largely. The method is a matter of custom. I do not roll all my pieces the same size. Where I have direct access to filling I want a large piece; or, if the borders are getting too low, I want smaller pieces, to bring them higher than center. It has been my custom to roll gold with my fingers. I do not think any foreign matter can get into it that way; my fingers are always dry. Whether you roll it into a cylinder or have it perfectly flat, it is the same; the gold is just as cohesive, and it would make very little difference, so far as air is concerned, whether it is in a sheet form or roll. The air would not have a very good chance after I get done with it; the air would all be excluded before I put my next piece in place, so I don't think it makes a great deal of difference.

The essayist speaks of stuffing cavities; there is a great deal of that done. You have all seen fillings of that kind. They may take a beautiful finish and look perfect, and yet not be adapted to the walls of the cavity.

I have in mind an operator who has now passed away. He used the electric mallet and lost a large percentage of his fillings because they were hard in the center and always leaking around the borders. Now this was either due to too much malleting or the pluggers passed through the gold instead of carrying it forward. If the plugger is too fine, or the gold too light, the plugger passes through and the borders of the enamel are likely to be injured and decay take place in a short time, and yet the filling may be just as hard as plate gold.

Dr. Warboys: I made a little note here and have a question to ask on it. I ask it because I want information, not knowing anything about it. I would like to know if the sponge gold, or mat gold, needs less retaining form than the foil. From what I have heard in reference to these mat golds, all you have to do is to place it in the cavity and it will stick there. Now, why is this? Does it possess any more strength than foil?

Dr. Crouse: Yes; it does not need so much retaining form.

Dr. Hall: I think the essayist's idea was this, that it did not require so deep a retaining form in which to start the filling with the mat gold as with others. The peculiar form of the gold is such that as you press it into a cavity it does not slip around as the foil does. By using large pieces and large pluggers you can push it into place, and on further condensation it will spread into the angles or form and remain there. It requires no pits.

Dr. Crouse: I want to discuss for a minute or two the question of cohesive or non-cohesive foils. Now, all gold which is absolutely pure is cohesive, and that proposition can not be disputed. If it is non-cohesive it is coated with something or it is impure, and I have never used it in my practice. The gold which comes nearest being soft is Abbey's soft gold foil. I can have gold just as soft as I want it now, or I can anneal it and make it cohesive. I can have gold absolutely non-cohesive and make it cohesive by subjecting to red heat.

Before I forget it, the essayist left out one important preparation of gold, and that is platinized gold. R. S. Williams made it before he died. It was No. 60. At present I am unable to get it in the market. It works very well and you make so much better appearing filling with it; the color will be such as to average clear out of sight and will last two or three years longer than any gold filling. It is harder than gold. It is made of a sheet of pure gold with a layer of platinum, and this is run through red hot rollers. I think the art is now lost. I know of no gold workers who are making it. They are giving us strips of platinum and gold. If you heat it you bring the platinum to the surface and its cohesive properties are lost. In my mind it is very much needed in the profession.

About one-half of the gold I now use is non-cohesive. I am not old, but I am old enough to be still using cylinders. I believe I can get better results by their use. I roll the cylinders myself, or have my assistant make them of different sizes for different cavities. When the cylinder sets in the bottom of the cavity I want it to extend over the edge. I will have my filling made in this way, three-fourths done while you, with your cohesive foil, are just starting, and I will wager I can do it. I do not wish to do it to-morrow, however, because I want to go on to New York, but I will challenge any man on this proposition.

I use the cylinders of non-cohesive gold for two reasons—we get better adaptation and make the filling in shorter time. Nobody used heavy foil as much as I did at one time. There was never a book of gold which came into my office under No. 60. I used Nos. 60 and 120, and I never had fillings which were better made. But it takes to much time; it takes too long to start. Men who have made fillings by the use of the heavy foils have labored harder and not made as good fillings, and it has taken them longer.

I remember once Dr. Webb was to make a filling, illustrating the use of the electric mallet, he asked me to prepare the cavity while he was getting ready. He told me to put in some retaining pits. I told him he did not need any pits; make the filling without them, and while he was getting his mallet ready I started the filling and had it three-fourths filled before he was ready to commence. That is the way I have made money practicing dentistry, doing the best work in the least time.

I have had the craze on crystal gold, but I am now out of it and do not think I will ever catch it again. I do not think, all things considered, you can make as good a filling in as short a time as with foil.

In reference to the matrix, I started filling teeth with the matrix, but have given it up; it is all right with amalgam. I want a good lateral pressure in making gold fillings, and you can not get it with the matrix.

QUESTION: Why with amalgam, and not gold?

DR. CROUSE: Because amalgam shifts under pressure and gold will not. Amalgam should be made with the matrix and much pressure used to insure its packing against borders. Dr. Black has come out with a machine for testing the force exerted by different ones using hand pressure, and it is amazing to find how little force a man can make with hand pressure; ten pounds is a big average. It was discovered that students did not use enough force to insure any kind of success with this filling. How much more force is required to make good gold fillings.

DR. PARKER: I commenced very early in my practice to use Abbey's foil, and I can confirm what the Doctor has just said, that the Abbey foil was not a pure gold. When we began the use of cohesive foil I found I could put the cohesive foil into the flame and it would have a different color from the other, and I think the secret of its being non-cohesive was that it was heavily coated with some preparation, so that probably an oxide formed on its surface rendered it non-cohesive.

QUESTION: I would like to ask Dr. Crouse how he makes cohesive gold non-cohesive?

DR. CROUSE: By putting it into a jar with a piece of cotton saturated with ammonia. By heating it again it will become cohesive.

QUESTION: What is the advantage of non-cohesive foil?

DR. CROUSE: The advantage is you can condense it more readily, that is, in larger masses, without its choking up; for example, in large cavities I always start with No. 3 non-cohesive gold, and sometimes make two sheets into one cylinder, but this is only for very large cavities; a sheet of No. 3 gold made into one cylinder is large enough. If I should take the same quantity of cohesive gold and undertake to drive it into a cavity, it would clog or bridge over and not fill in the cavity. Sometimes in using non-cohesive gold it does not go into the right place in the cavity, I take it out and reroll it. It does not weld. I do not think it is necessary to weld the gold at the base of the cavity, I think it answers many times better not to weld it. I have made fillings with cohesive gold in the last few years which have not lasted, while others I made years ago with the other gold are giving good service to-day.

QUESTION: You do not use the mallet very much with these cylinders do you?

DR. CROUSE: I do not use mallet at the start, but I finish with cohesive gold and then mallet.

DR. RICKS: I saw a filling yesterday which was made with soft foil by me about forty years ago next month; it was wedged in. We did not have any cohesive foil at that time, at least I had never used any of it. I think the first time I ever saw a filling made with cohesive foil was in a clinic given by Dr. Watling—I had never seen anything of it up to that time.

We used to wedge our fillings in with soft foil; we would put the cylinders in so as to extend above the margin of the cavity; we never thought of making a contour filling because we knew nothing about it; we did not expect to ever weld the foil, simply wedged it in.

DR. HOFF: I want to say a word in reference to Dr. Crouse's use of the non-cohesive foil in cylinders which enables him to do a great deal of work in a very short time, and do it well. I have no doubt he can do that, and I have long thought that I would learn the method of filling teeth with cylinders, because next to No. 60 foil, or in combination with it, I think the cylinder filling method is ideal. I do not approve, however, of Dr. Crouse's method of using it. In making a gold filling I

want first a firm anchorage or seat, and I put my first piece of gold where I want it to stay. If he makes no more complete condensation of his first piece so that it is possible to take it out and unroll it and again place in the cavity, I can not see what assurance he has of his margin contacts. I should think it would slip and slide around in the cavity indefinitely. In preparing a cavity I want it of such shape that every piece of the filling will be held in by a properly shaped cavity wall, and I can not see how a non-cohesive foil filling could be placed in a cavity as prepared by Dr. Crouse, as I understand it, without a matrix. I might, if I had a cavity with straight sides in the occlusal surface of a molar, pack the gold so that it would remain.

Dr. Crouse: I do not mean to say I have no retaining shape to my cavities, but I want a perfectly flat base and no retaining pits of any kind.

Dr. Hoff: It is not the way you pack the gold, but the kind of a foundation you make that I refer to particularly. I don't think that there can be anything securely built on top of your foundation. The most important part of any filling is the point of occlusal contact, not the side walls of the filling, but at the point where the strain comes on the filling at the occlusal surface. The heavy foil is ideal placed there, but unless you have something solid to build it upon the filling will be worthless; it is like building upon a rubber cork, it will give and render it impracticable to make secure joints. My idea of filling is this, the first piece—and in fact every piece—of gold I put into the cavity I want to stay exactly where placed with no after shifting of position. I use starting pits, I undercut these pits, but I do not make them very deep, simply starting points, they are not intended to hold the filling in. They are usually round holes made by a flat-pointed drill or an inverted cone bur, the pit is drilled only slightly deeper than the diameter of the drill or bur used. The first piece of gold when packed into such a pit is going to remain there, and when the next piece is added it does not shift at all, but coheres to the first piece of gold, and a filling built upon such a foundation will have secure joints and withstand the strain of mastication with slight anchorage undercuts or grooves. In reference to the matrix I know that some of our best operators do not approve of the matrix. I had a conversation with Dr. Black on that subject last summer; he does not use the matrix. I think I can make just as good a gold filling with the matrix, and in many cases a better one than can be made without; of course, the cavity needs special preparation.

